# Sustained complete remission in chemotherapy-refractory advanced lung adenocarcinoma with bevacizumab and iodine-125 seed implantation: A long-term case report

**DOI:** 10.1097/MD.0000000000047018

**Published:** 2026-01-09

**Authors:** Dan Ma, Nuo Jin, Zhuo Song, Chaochao Jin, Enfeng Song

**Affiliations:** a Department of Traditional Chinese Medicine, The Affiliated Dongguan Songshan Lake Central Hospital, Guangdong Medical University, Dongguan, China; b Sterilization and Supply Department, Huangshi Central Hospital, Huangshi, China; c Department of Radiotherapy, Air Force Medical Center, Air Force Medical University, Beijing, China; d Department of Oncology, Huangshi Traditional Chinese Medicine Hospital, Huangshi, China; e Department of Traditional Chinese Medicine, Renmin Hospital of Wuhan University, Wuhan, China.

**Keywords:** advanced lung adenocarcinoma, bevacizumab, brain metastases, case report, iodine-125 seed implantation

## Abstract

**Rationale:**

The introduction of small-molecule tyrosine kinase inhibitors and immune checkpoint inhibitors has transformed therapeutic strategies for lung adenocarcinoma, significantly improving the survival of eligible patients. However, the prognosis remains poor for those lacking actionable driver mutations and presenting with brain metastases, who typically exhibit a median survival of <6 months.

**Patient concerns:**

After the initial resection (November 2016) and progression after adjuvant paclitaxel/cisplatin chemotherapy, the patient developed brain and pulmonary metastases.

**Diagnoses:**

Lung adenocarcinoma (cT3N0M1c, AJCC 8th edition) with synchronous brain and pulmonary metastases molecularly characterized as epidermal growth factor receptor- and anaplastic lymphoma kinase-negative.

**Interventions:**

Complete remission was attained through a combined approach: whole-brain radiotherapy (WBRT) for intracranial lesions, CT-guided iodine-125 (^125^I) seed implantation for lung metastases, and maintenance bevacizumab (BEV).

**Outcomes:**

At the latest follow-up, the patient maintained complete remission for over 92 months, with a Karnofsky performance status of 100, demonstrating unprecedented long-term survival.

**Lessons:**

This case demonstrates the potential synergistic efficacy of whole-brain radiotherapy, ^125^I brachytherapy, and antiangiogenic therapy in advanced epidermal growth factor receptor/anaplastic lymphoma kinase wild-type lung adenocarcinoma, providing a novel salvage strategy for chemotherapy-refractory metastatic disease. Prospective clinical trials are warranted to validate the survival benefits of this multimodal approach in molecularly unselected, high-risk populations.

## 1. Introduction

Lung cancer remains the leading cause of cancer-related mortality worldwide and is responsible for 20% of all cancer deaths.^[1,2]^ Non-small cell lung cancer (NSCLC) constitutes 85% of NSCLC cases,^[3,4]^ with lung adenocarcinoma (LUAD) representing the predominant subtype, accounting for 47% of western and 55% to 60% of Asian NSCLC cases.^[5]^ Despite transformative advances in targeted therapies and immune checkpoint inhibitors, long-term responses remain elusive in 50% to 60% of patients with LUAD lacking actionable driver mutations.^[6]^ This is particularly evident in advanced LUAD with brain metastases (BM), where the median survival is only 6 to 7 months.^[7]^ Current BM management relies on multimodal approaches, including whole-brain radiotherapy (WBRT), stereotactic radiosurgery, and systemic therapies; however, WBRT alone extends survival to just 4.2 months in unselected LUAD.^[8]^

Bevacizumab (BEV), a vascular endothelial growth factor (VEGF)-targeting monoclonal antibody, disrupts tumor angiogenesis and normalizes aberrant vasculature, potentially enhancing radiotherapy efficacy.^[9]^ Recent meta-analyses have demonstrated that BEV combined with radiotherapy improves objective response rates (48% vs 28%) and reduces peritumoral edema in NSCLC BM.^[10]^ However, its role in epidermal growth factor receptor (EGFR)/anaplastic lymphoma kinase (ALK) wild-type LUAD remains to be explored.

Iodine-125 (^125^I) brachytherapy delivers continuous low-dose radiation (0.4–1.0 Gy/h), offering precise tumor control with minimal toxicity.^[11]^ Emerging evidence supports its utility in oligometastatic NSCLC, with local control rates exceeding 70%.^[12]^ However, data on ^125^I + BEV combinations in driver-negative LUAD are sparse, despite mechanistic synergies; BEV-induced vascular normalization may enhance ^125^I radiosensitivity.^[13]^

Here, we present a paradigm-challenging case of advanced EGFR/ALK wild-type LUAD that achieved sustained complete remission (CR) through innovative multimodal therapy. Following curative-intent resection (November 2016) and subsequent development of BM and pulmonary metastases after platinum-doublet adjuvant chemotherapy, the patient demonstrated an exceptional response to a 3-pronged approach: WBRT for intracranial control, image-guided ^125^I brachytherapy for pulmonary lesions, and maintenance BEV therapy. Remarkably, the patient maintained a radiologically confirmed CR with an excellent performance status (Karnofsky performance status 100) at >92 months of follow-up. This unprecedented outcome provides clinical evidence supporting the potential synergistic effect between tumor vasculature normalization (via BEV) and enhanced radiosensitivity (via ^125^I) in chemotherapy-refractory LUAD, suggesting a novel therapeutic strategy worthy of prospective investigation.

## 2. Materials and methods

### 2.1. Information about the patients, clinical findings, and timeline

Our study results are reported in the CARE guidelines.^[14]^ A 65-year-old male heavy smoker (45 pack-year history) underwent radical left upper lobectomy with systematic lymph node dissection on November 29, 2016, for a 7.0 cm × 5.0 cm × 5.0 cm pulmonary mass (Surgical records can be found in Supplementary Material 1, Supplemental Digital Content, https://links.lww.com/MD/R108). The patient had no family history of cancer. Histopathological examination of the resected specimen confirmed invasive adenocarcinoma (pT3N0M0, stage IIB according to the AJCC 8th edition^[15]^) with clear surgical margins. A comprehensive lymph node evaluation revealed no metastatic involvement in any of the 24 dissected nodes (0/24). Immunohistochemical profiling demonstrated strong positivity for TTF-1 (diffuse+), napsin A(+), P53 (mutant pattern+). Focal expression: p63 (scattered+), ROS1 (weak+). Negative markers: CK5/6(−), P40(−), ALK (D5F3) (−). High proliferative index: Ki-67: 70%. Molecular characterization using ARMS-PCR with fluorescence detection excluded common driver mutations: EGFR wild-type (exons 18-21), ALK rearrangement-negative. (The postoperative pathological report and genetic test report are shown in Supplementary Materials 2 and 3, Supplemental Digital Content, https://links.lww.com/MD/R108.) Preoperative staging workup, including contrast-enhanced chest/abdominal CT, brain MRI with gadolinium, and whole-body bone scintigraphy, revealed no evidence of distant metastasis. He was asymptomatic at the time of diagnosis.

### 2.2. Informed consent and ethics clearance

Written informed consent was obtained from the patients on a free and voluntary basis, and clinical data were fully anonymized. The Clinical Research Ethics Committee approved the study protocols and the Ethics Committee of the Huangshi Hospital of Traditional Chinese Medicine, Hubei, China (HSZYPJ-2024-001-01). This study was conducted in accordance with the Declaration of Helsinki and its modifications.

### 2.3. Diagnostic assessment and therapeutic intervention

The diagnosis of stage IV LUAD was confirmed through an integrated multimodal evaluation. Postoperative histopathological analysis revealed a primary pulmonary adenocarcinoma with a T3 classification. Whole-body contrast-enhanced CT and brain MRI demonstrated synchronous pulmonary and multifocal BM. Molecular profiling by ARMS PCR confirmed the EGFR and ALK wild-type status. Final staging was designated as cT3N0M1c, according to the AJCC Cancer Staging Manual (8th edition).^[15]^

**Figure 1.**
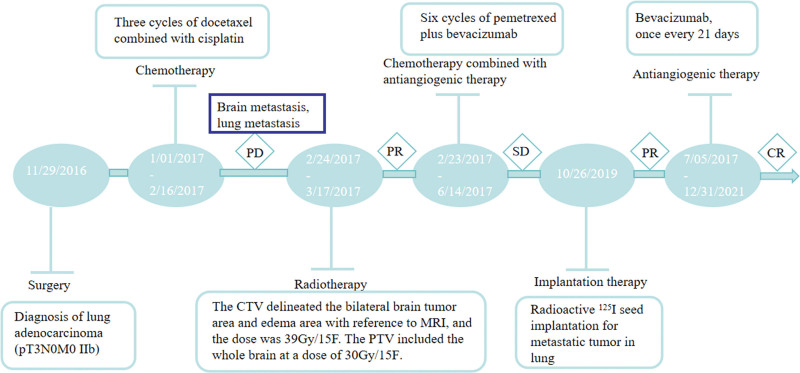
Schematic diagram of the patient’s treatment timeline.

**Figure 2.**
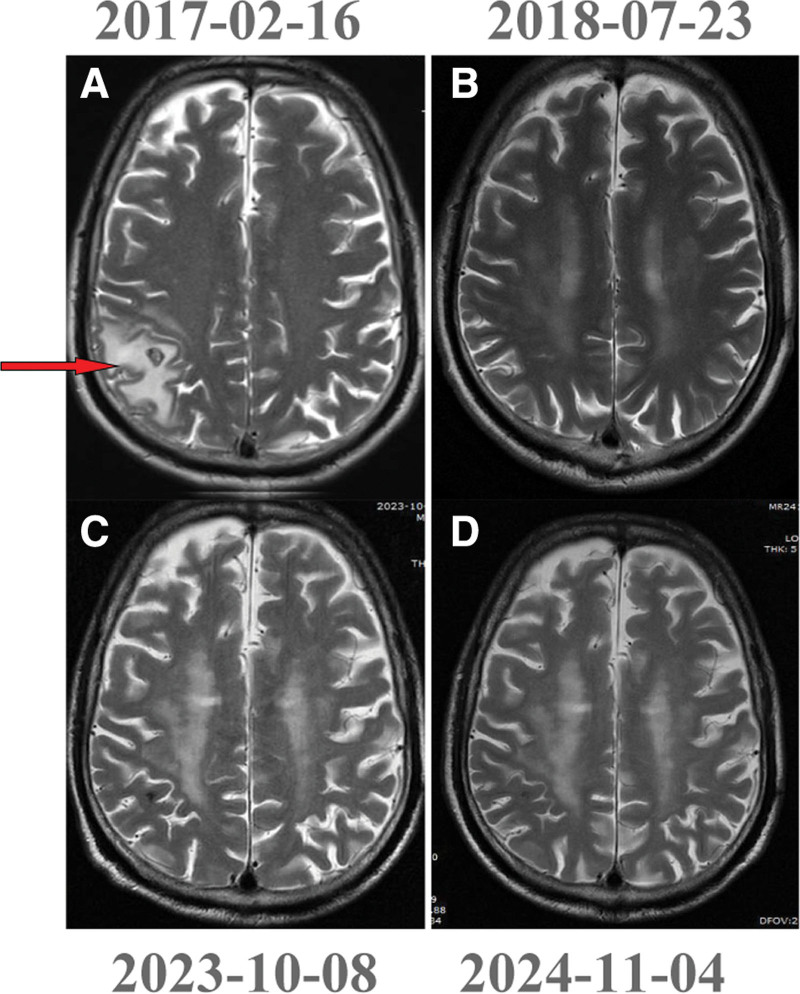
Brain MRI images. (A) Brain metastasis and edema after 3 postoperative cycles of paclitaxel plus cisplatin. (B–D) No recurrence after 16, 79, and 92 months of whole-brain radiotherapy combined with bevacizumab. MRI = magnetic resonance imaging.

The treatment timeline is shown in Figure 1. Following surgical resection with a final pathological staging of pT3N0M0 (stage IIB according to the AJCC 8th edition) and confirmation of negative EGFR/ALK mutation status, the patient received 3 cycles of adjuvant chemotherapy (docetaxel 75 mg/m² + cisplatin 75 mg/m²) from January to February 2017, in accordance with the NCCN guidelines at the time. Five days after chemotherapy, the patient developed grade 2 neurological symptoms (CTCAE v5.0^[16]^). Brain MRI revealed a 2.4 cm right parietal metastasis with significant perilesional edema (Fig. 2A), prompting immediate intervention with: Symptomatic management: mannitol (20%, 125 mL q6h) for cerebral edema; and radiation therapy: Hippocampal-sparing WBRT (30 Gy/15 fractions) with simultaneous integrated boost (39 Gy/15 fractions) to gross tumor volume. Systemic therapy: BEV (15 mg/kg) combined with pemetrexed chemotherapy was initiated on February 23, 2017, based on contemporary NCCN guideline recommendations for EGFR/ALK wild-type metastatic LUAD. The patient subsequently completed 6 cycles of pemetrexed (500 mg/m²) plus bevacizumab (15 mg/kg), followed by maintenance bevacizumab until December 31, 2021.

The decision to continue BEV maintenance until December 31, 2021, was based on: sustained intracranial response observed on serial MRI scans. Apart from enlargement of the lung lesions, no new lesions or metastases were observed in other parts of the body. Participation in a patient assistance program that provided BEV without cost, and absence of significant treatment-related toxicities (only grade 1 hypertension managed with captopril).

**Figure 3.**
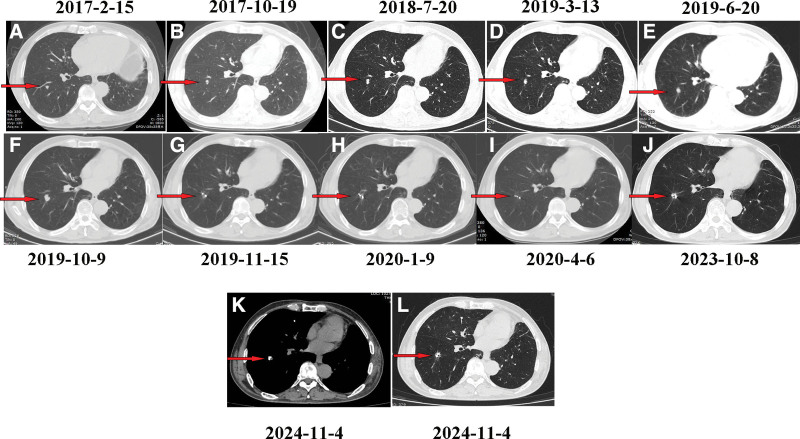
Lung CT images. (A) February 15, 2017: A new metastatic lesion (arrow) in the right lower lobe detected after 3 cycles of paclitaxel plus cisplatin chemotherapy. (B) October 19, 2017: The metastatic lesion (arrow) remains stable in size after 6 cycles of pemetrexed plus bevacizumab. (C) July 20, 2018: Continued stability of the lesion (arrow) was observed after 12 months of bevacizumab maintenance therapy. (D) March 2019: The lesion (arrow) measuring 12 mm × 10 mm. (E) June 20, 2019: The lesion (arrow) remains stable (12 × 10 mm) after 23 months of bevacizumab treatment, showing no significant change from the previous scans. (F) October 9, 2019: Oligoprogressive disease is noted, with enlargement of the lesion (arrow) to 15.2 mm × 14.6 mm after 27 months of bevacizumab, before ^¹²⁵^I brachytherapy. (G) November 15, 2019: 1 month after ^¹²⁵^I implantation, no recurrence is observed. (H) January 9, 2020: 3 months postimplantation, no recurrence was observed. (I) April 6, 2020: At 6 months postimplantation, there was no evidence of recurrence. (J) October 8, 2023: No local recurrence was evident 48 months after brachytherapy. (K) November 4, 2024: Mediastinal window view 61 months postimplantation shows no recurrence. (L) November 4, 2024: Lung window view at 61 months postimplantation confirms no recurrence. CT = computed tomography.

Serial surveillance with chest CT and brain MRI performed every 3 months revealed no intracranial recurrence (Fig. 2B–D) and stable pulmonary lesions (Fig. 3A–C). However, follow-up imaging in October 2019 demonstrated slight enlargement and increased solid components of the lung metastases. Specifically, the metastasis in the right lower lobe increased in size from 12 mm × 10 mm on March 13, 2019, to 15.2 mm × 14.6 mm in October 2019 (Fig. 3D–F). The tumor marker carcinoembryonic antigen remained within normal limits, and no relapse was detected elsewhere in the body. A percutaneous biopsy of the lung mass was performed on October 26, 2019, with pathology confirming recurrent lung adenocarcinoma. This was followed by CT-guided implantation of 6 Iodine-125 radioactive seeds (0.6 mCi per seed). Subsequent chest CT examinations from January 9, 2020 to November 4, 2024, showed no evidence of local recurrence (Fig. 3H–L).

### 2.4. Treatment and follow-up

At the most recent reexamination (November 4, 2024), comprehensive evaluation revealed sustained complete radiographic response (Figs. 2D and 3K, L); normal serum tumor markers, no evidence of local recurrence or distant metastasis, and treatment-related adverse events including grade 1 hypertension (150/90 mm Hg) and captopril (25 mg BID) until BEV discontinuation. Hypothyroidism: Well-controlled with levothyroxine (100 μg/d). No grade ≥2 toxicities (CTCAE v5.0).

## 3. Results

The patient maintained an excellent functional status (Karnofsky performance status 100) with normal hepatic and renal function at the most recent follow-up on March 27, 2025. The patient denied any symptoms of discomfort and reported no dizziness, headache, coughing episodes, or chest pain.

## 4. Discussion and conclusions

Our case presents the initial report of a durable response and an overall survival (OS) exceeding 92 months in a patient with advanced LUAD with a significant tumor burden, including lung metastases and BMs, who tested negative for EGFR exons 18/19/20/21 and ALK rearrangements. This was significantly better than the median progression-free survival (PFS; 8.0 months) and OS (13.5 months) for BMs in NSCLC treated with BEV combined with WBRT in the existing literature.^[10]^

LUAD is the foremost contributor to global cancer mortality, with population-based registries documenting a persistently poor 5-year survival rate of 14% for advanced-stage disease.^[16]^ Contemporary diagnostic patterns reveal that approximately 70% of LUAD cases present with locally advanced or metastatic disease at the initial diagnosis, necessitating multimodal systemic therapies.^[17]^ Although platinum-based doublet chemotherapy remains a cornerstone of treatment, its clinical utility is constrained by moderate response rates (15–35%) and considerable toxicity profiles.^[18]^ Over the past 2 decades, the development of targeted therapy and utilization of immunotherapy have led to significant advances in the treatment of LUAD. The precision oncology era has introduced molecularly targeted agents and immune checkpoint inhibitors, revolutionizing care for molecularly defined subsets. However, approximately 50% to 60% of patients with LUAD lack actionable driver mutations.^[6]^ It is a recognized fact that LUAD patients without driver gene mutations often choose to be treated with immunotherapy or combination chemotherapy, but overall cure rates and survival rates remain low, 5-year overall survival is <15%, especially for metastatic disease.^[19]^

Notably, programmed death ligand-1 (PD-L1) expression was not routinely assessed in clinical practice when a patient was first diagnosed with LUAD in 2016. Owing to the sustained complete response achieved with combination therapy of ^¹²⁵^I brachytherapy and bevacizumab, along with the absence of disease recurrence, no additional invasive biopsy has been performed to evaluate PD-L1 expression levels thus far. If disease progression occurs in the future, we will prioritize repeat biopsy and conduct comprehensive molecular profiling, including PD-L1 expression, to guide subsequent treatment decisions. If PD-L1 expression is positive, immunotherapy may be considered, whereas if PD-L1 remains negative, bevacizumab or other antiangiogenic therapies may be reinitiated.

BM remains a significant problem in patients with lung cancer. The median 1-, 2-, and 3-year OS rates for patients with BM were 6 months and 29.9%, 14.3%, and 8.4%, respectively.^[20]^ The therapeutic armamentarium has expanded from WBRT and surgery to encompass stereotactic radiosurgery, targeted therapies, and immunotherapies, often used sequentially or in combination.^[21]^ In this case, the combination of WBRT and BEV in treating BM resulted in promising CR, which is consistent with the results in the literature.^[22,23]^

For advanced LUAD with a single lung metastasis, the first treatment choice recommended by the NCCN guidelines is surgery.^[24]^ Considering the patient’s previous left lung cancer surgery, left upper lobectomy, poor pulmonary function, and recurrent non-peripheral metastasis, a comprehensive assessment of the patient chose a reliable and less expensive radioactive particle implantation therapy to achieve simple and effective inexpensive treatment. The patient had poor postoperative pulmonary function. External beam radiotherapy may have side effects, such as radiation pneumonitis and radiation esophagitis, while particle therapy in brachytherapy can effectively target pulmonary metastases.

Brachytherapy with seed implantation has demonstrated promising efficacy in the treatment of various types of locally recurrent tumors. For patients with locally recurrent rectal cancer who have previously undergone external beam radiation therapy or surgery, ^125^I seed implantation represents a safe and effective salvage treatment option.^[25]^ A case indicated that ^125^I seed implantation could be an alternative local therapy for metastatic pheochromocytoma in the lungs.^[25]^ In recent years, several clinical studies have confirmed that radioactive seed implantation has a positive effect on metastatic lung cancer.^[26,27]^ A meta-analysis of 19 clinical trials of radioactive seed therapy for advanced NSCLC showed that radioactive seed therapy showed promising efficacy and acceptable toxicity in patients with locally advanced NSCLC.^[28]^

Angiogenesis is a fundamental hallmark of cancer progression, making it an attractive therapeutic target for the treatment of advanced LUAD. Among the antiangiogenic agents, BEV has demonstrated particular clinical utility in the management of BMs from LUAD. As a humanized monoclonal antibody targeting VEGF, BEV exerts dual mechanistic effects: inhibition of tumor angiogenesis and reduction of vasogenic edema through vascular normalization. The clinical efficacy of BEV in LUAD patients with BMs is supported by a comprehensive meta-analysis of 17 studies, which demonstrated an intracranial PFS of 8.0 months and an OS of 13.5 months in this patient population.^[10]^ Notably, our case report revealed an exceptional outcome, with WBRT combined with BEV maintenance therapy achieving an ongoing CR exceeding 96 months. This remarkable result suggests that BEV maintenance therapy may substantially improve PFS in patients with advanced LUAD and BMs who lack common driver mutations.

BEV combined ^125^I particle implantation therapy can achieve such a long PFS and tumor-free and asymptomatic survival, with the following mechanisms: BEV enhances the radiosensitivity of ^125^I particles through vascular normalization^[29]^; the synergistic effect of sustained low-dose radiation and antivascular therapy overcomes common chemotherapy resistance^[30]^; and the spatio-temporal cooperative effect of local control and systematic therapy. This combination strategy offers a potential breakthrough treatment option for patients with driver-negative advanced LUAD; however, it still needs to be validated in prospective studies.

Furthermore, our case report invites speculation regarding the potential biological mechanisms underlying this exceptional response. In addition to its established antiangiogenic effects, bevacizumab is known to modulate the tumor immune microenvironment. Recent advances in oncological research have highlighted the critical prognostic value of immune-inflammatory and nutritional biomarkers in solid tumors, including colorectal and breast cancers.^[31–33]^ In the context of our findings, it is plausible that the combination of brachytherapy and BEV may have fostered a favorable immune milieu, potentially involving the alteration of such systemic biomarkers, which could be measured in future studies to identify predictive signatures for this combination therapy.

The mechanistic basis of the observed synergy warrants further investigation using modern profiling techniques. Cutting-edge transcriptomic and single-cell analyses in breast cancer have recently identified immune regulatory genes, such as *IL27RA* and *TMEM71* as novel therapeutic indicators and potential targets.^[34,35]^ We hypothesized that similar mechanisms may play a role in sensitizing tumors to antiangiogenic therapy and radiation in LUAD. Future research should prioritize profiling serial tissue and liquid biopsies from patients receiving this combination to identify and validate analogous predictive immune signatures or cellular states that can guide optimal patient selection and identify new therapeutic targets.

Despite the encouraging outcomes observed in this case, several limitations must be acknowledged. First, as a single-case report, our findings inherently lack statistical power and generalizability of prospective clinical trials. The exceptional response observed here may not be replicable across a broader, more heterogeneous patient population. Second, the applicability of this combined modality approach (^¹²⁵^I brachytherapy and sustained bevacizumab) could be constrained by significant barriers, including the requirement for specialized interventional radiology expertise for seed implantation, potential cost and accessibility issues of long-term bevacizumab therapy, and the need for meticulous patient selection. Ideal candidates are likely those with oligoprogressive disease and a good performance status, as in our patient. Finally, although this strategy proved highly successful in a treatment-refractory, driver mutation-negative setting, its efficacy relative to modern first-line options, such as immunotherapy combinations, remains undefined and warrants further investigation in larger, controlled studies.

Looking forward, validating the efficacy of this combined modality approach (^¹²⁵^I brachytherapy and BEV) will require prospective trials with carefully defined patient selection criteria. Based on our experience, ideal candidates would likely include individuals with *EGFR/ALK* wild-type advanced LUAD exhibiting an oligoprogressive disease pattern (e.g., 1–3 metastatic lesions) following front-line therapy, who maintain a good performance status (ECOG 0-1). Furthermore, biomarker-driven selection should be explored; for instance, tumors with high VEGF expression, specific immune microenvironment profiles, or those resistant to immunotherapy may derive particular benefits. The feasibility of safely performing image-guided brachytherapy, determined by the lesion size, location, and proximity to critical structures, is also a critical practical criterion. Ultimately, defining the optimal biological and clinical context for this strategy is essential for translating these promising results into broader clinical practice.

### 4.1. Patient’s perspective

Following 3 cycles of postoperative chemotherapy (docetaxel plus cisplatin), the patient developed BM with refractory cerebral edema that was poorly responsive to mannitol and dexamethasone. The subsequent combination of WBRT and BEV partially alleviated vertigo and vomiting. After completing 6 cycles of pemetrexed-bevacizumab regimen followed by 2.5-year BEV maintenance therapy, along with ^125^I brachytherapy for oligometastatic pulmonary lesions, all follow-up examinations demonstrated excellent clinical outcomes. I remained asymptomatic with normalized imaging and laboratory findings. I am profoundly grateful to my medical team for timely WBRT and brachytherapy that controlled metastatic progression and sustained tumor suppression through BEV maintenance. Their precise therapeutic strategy has granted me renewed life.

## Acknowledgments

We thank the patient for his cooperation and the CT imaging physician for their help during the particle implantation procedure. We are grateful for the financial support provided by the Key Research and Development Program.

## Author contributions

**Conceptualization:** Enfeng Song.

**Investigation:** Dan Ma, Nuo Jin, Zhuo Song, Chaochao Jin.

**Writing – original draft:** Dan Ma, Nuo Jin, Zhuo Song, Chaochao Jin.

**Writing – review & editing:** Enfeng Song.

## Supplementary Material


