# Supplementary material for: Sustained complete remission in chemotherapy-refractory advanced lung adenocarcinoma with bevacizumab and iodine-125 seed implantation: A long-term case report
Source: Medicine (Baltimore). 2026 Jan 9;105(2):e47018. doi: 10.1097/MD.0000000000047018 (PMC12795093; doi:10.1097/MD.0000000000047018)

Supplementary Material 1. Intraoperative and Surgical Specimen Records.
This file contains the official surgical record from the radical left upper lobectomy procedure performed on November 29, 2016. It documents key operative details, including the dissection of lymph nodes. Additionally, it includes a photograph of the resected gross specimen, showing the pulmonary mass measuring 7.0 × 5.0 × 5.0 cm.


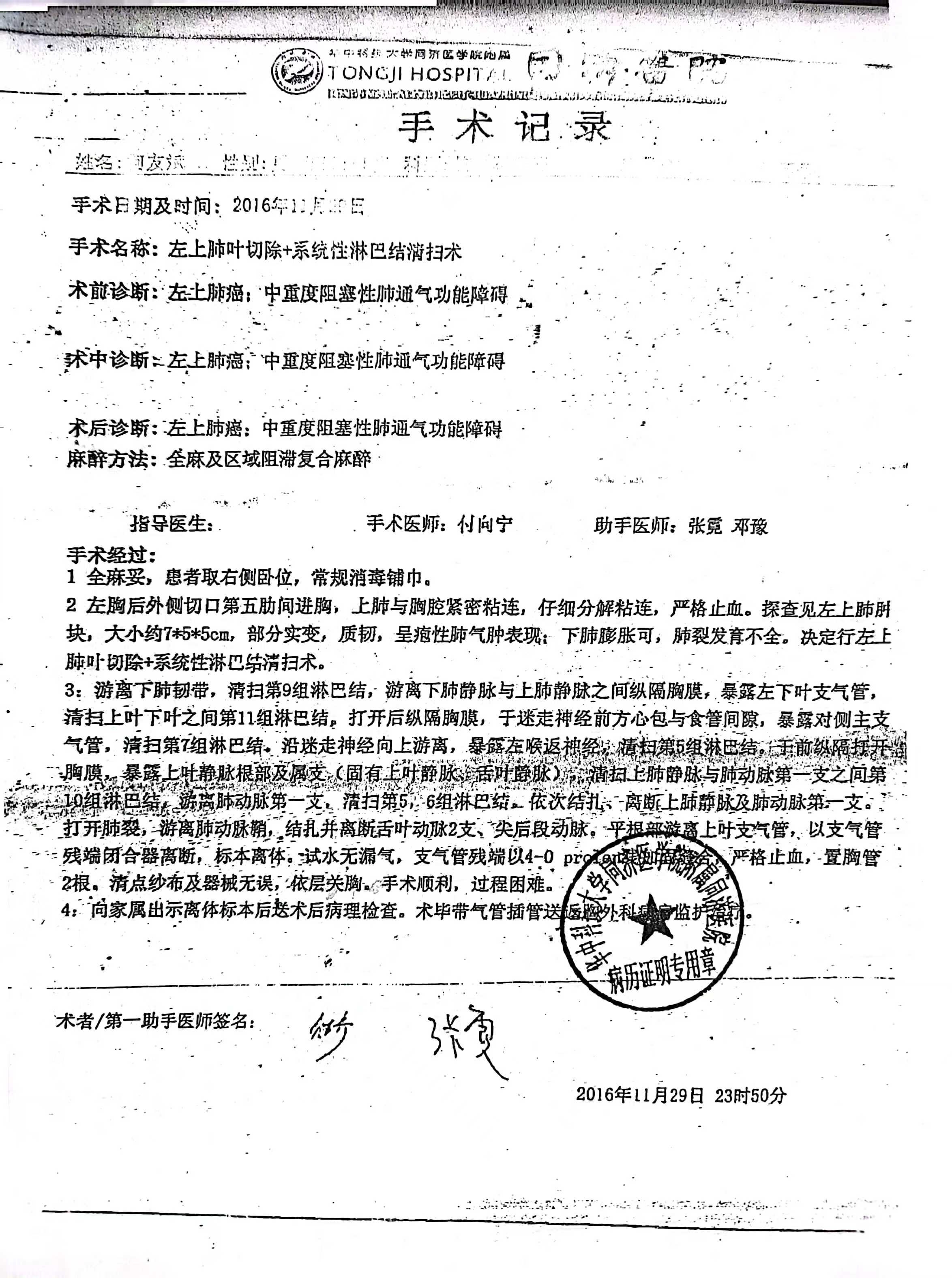


Supplementary Material 2. Postoperative Pathological Diagnosis Report.
This document is the complete histopathological report for the resected lung tumor. It confirms the diagnosis of invasive adenocarcinoma (pT3N0M0, Stage IIB, AJCC 8th edition) with clear margins and no metastatic involvement in 24 dissected lymph nodes (0/24). The report details the immunohistochemical staining profile, demonstrating positivity for TTF-1 and napsin A, a mutant pattern of P53, a high Ki-67 proliferation index (70%), and negativity for ALK (D5F3).


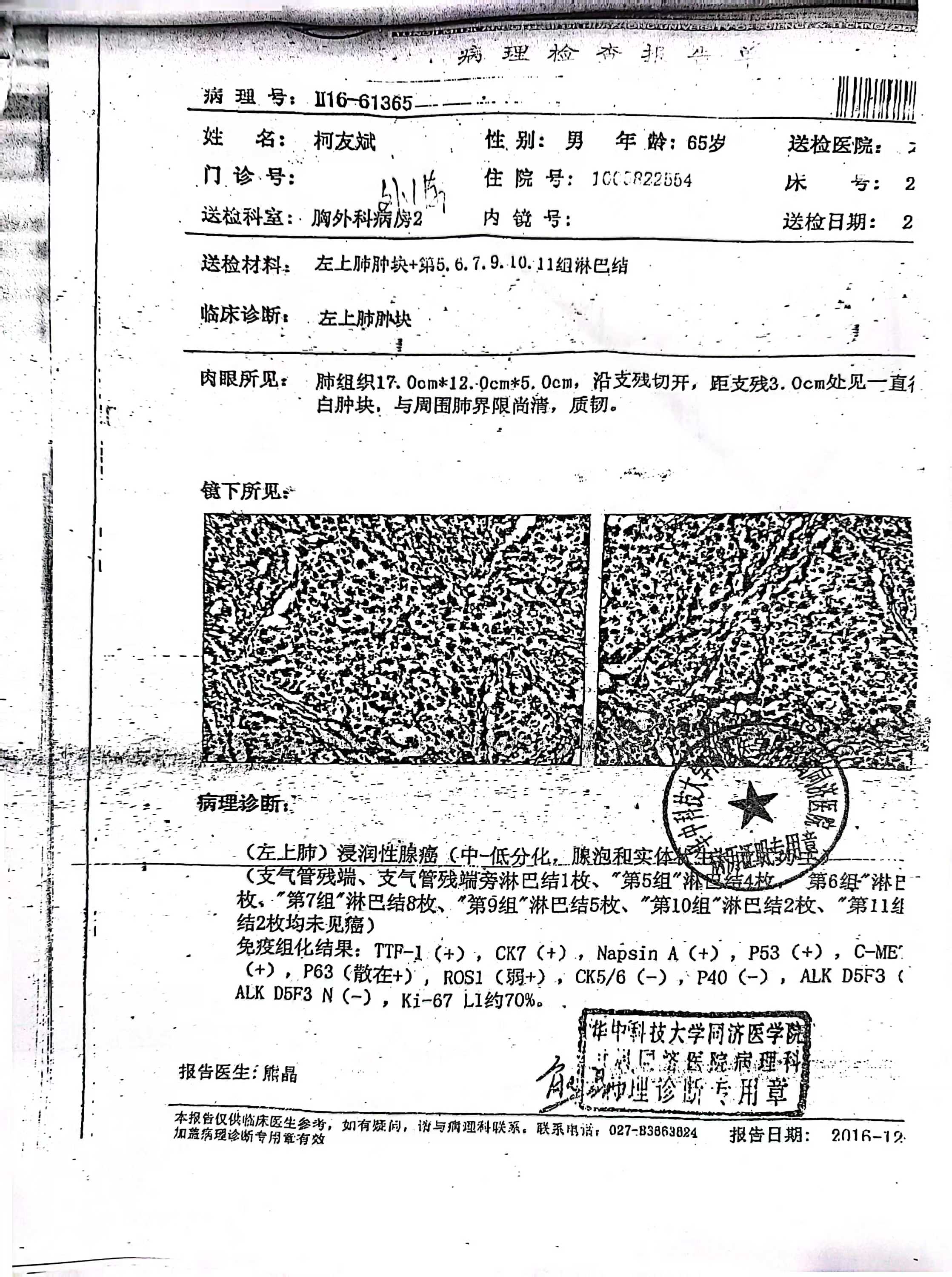


Supplementary Material 3. Somatic Mutation Analysis Report (ARMS-PCR).
This report presents the results of the molecular characterization performed using Amplification Refractory Mutation System Polymerase Chain Reaction (ARMS-PCR). The analysis confirms the tumor was wild-type for common driver mutations in the *EGFR* gene (exons 18-21).


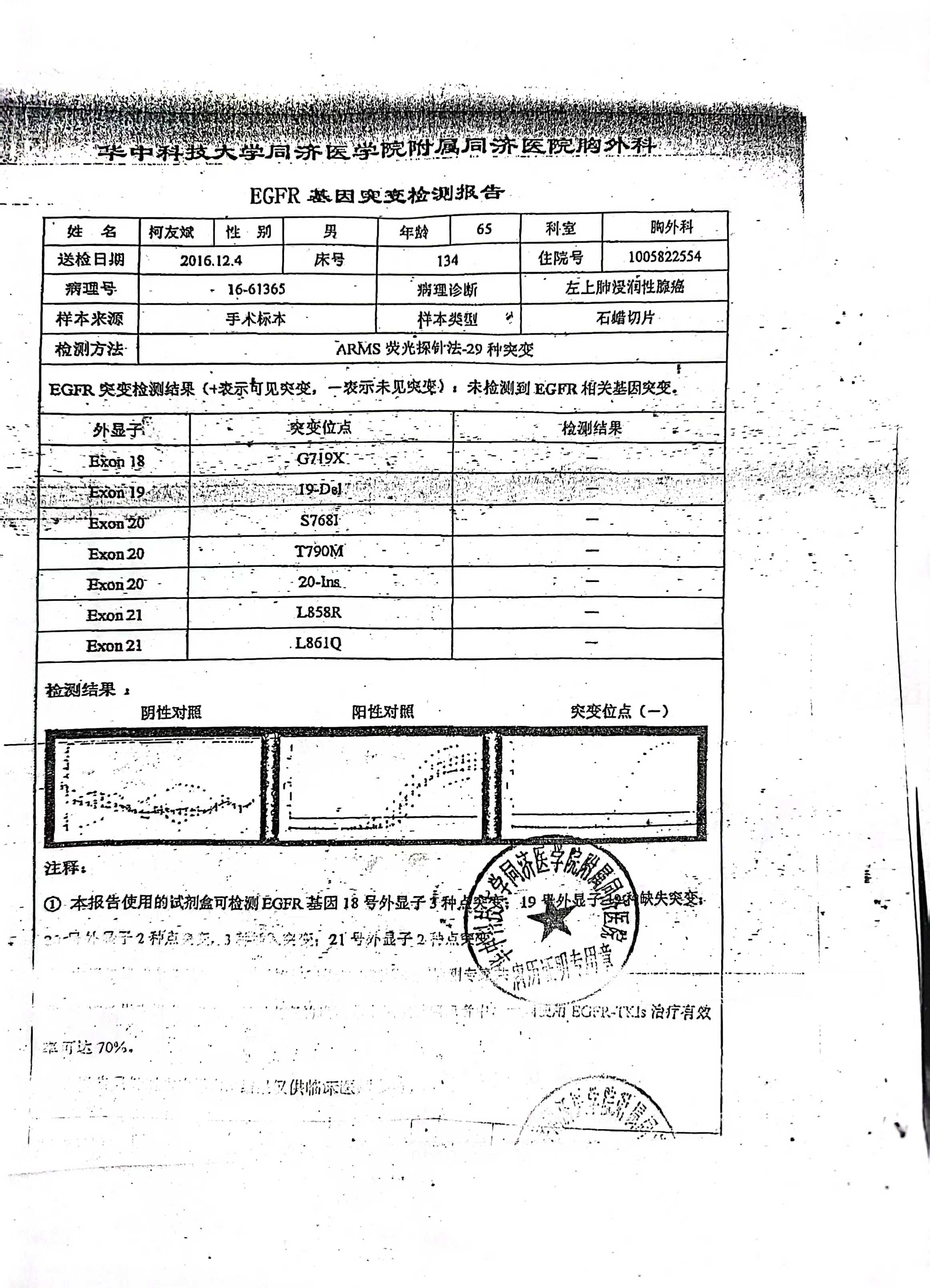

Supplement: Supplementary file 1 [file medi-105-e47018-s001.docx]
